# Induced erotomania by online romance fraud - a novel form of de Clérambault’s syndrome

**DOI:** 10.1186/s12888-024-05667-6

**Published:** 2024-03-20

**Authors:** Nasri Alotti, Peter Osvath, Tamas Tenyi, Viktor Voros

**Affiliations:** 1 Department of Psychiatry, Markusovszky Lajos University Teaching Hospital of Vas County, Szombathely, Hungary; 2 Department of Psychiatry and Psychotherapy, Medical School, University of Pecs, Pecs, Hungary

**Keywords:** Erotomania, de Clerambault’s syndrome, Online romance fraud, Mental disorders

## Abstract

**Background:**

Although the impact of internet usage on mental health is extensively documented, there is a notable scarcity of reports in the literature concerning internet-induced erotomania. Erotomania is a rare and likely underdiagnosed delusional disorder. It is characterized by an irrational belief held by the affected persons that someone of higher socioeconomic status harbor romantic feelings toward them. Here, we describe the psychopathology of erotomanic delusion induced by online romantic fraud in a female patient. Employing this case as a focal point, we illuminate novel aspects of erotomania that warrant attention and examination.

**Case presentation:**

We present a compelling case involving a 70-year-old married Caucasian woman diagnosed with medically controlled persistent depressive disorder for several years. The intricacies of her condition became evident as she became deeply engrossed in online profiles featuring the image of a renowned musician, inadvertently falling victim to an online romantic fraud. Subsequently, this distressing experience triggered the emergence of erotomanic delusions and a suicide attempt. The patient's history reveals an array of medical conditions and stressful life events, contributing to her vulnerability. The diagnosis of erotomanic delusional disorder, dysthymia, and mild cognitive impairment with cerebral vascular background was established. Treatment involved her previous antidepressant with low-dose risperidone, alongside supportive individual and group therapy. Her delusion showed remission four weeks later, prompting her discharge for outpatient follow-up. Although she retained some false beliefs, the intensity of the symptoms had notably diminished and her functionality improved.

**Conclusion:**

This case underscores the complex interplay between mental health, online activities, and the consequences of delusions, including suicidal thoughts, shedding light on the need for a comprehensive approach in addressing such challenging psychiatric scenarios.

## Background

In the era of the digital age, recent advancements in communication technology have profoundly impacted social interactions [1]. The popularity of these technologies is attributed to their characteristics of anonymity, openness, interactivity, convergence, decentralization, and globality [2–5]. However, these features have also provided a wide scope for online cybercrimes. One prevalent form of cybercrime is online romance fraud, which has become widespread with the advent of social media and dating apps [6]. Online romance fraud occurs when a criminal adopts a false online identity to deceive and gain love and trust [6]. Over the past few years, scammers have increasingly applied artificial intelligence (AI) to overcome language and cultural barriers, adding a new layer of sophistication to their deceptive tactics [7, 8]. The perpetrator strategically employs manipulation techniques to construct a deceptive facade, deftly fabricating an illusion of a romantic relationship with the unsuspecting victim, within the context of fraudulent activities. Their primary objective is financial exploitation, with little regard for the psychological harm inflicted on the victim as a potential consequence. Individuals contending with mental disorders face heightened risks of exploitation due to increased susceptibility and vulnerability. Specifically, those dealing with psychological and mental health challenges exhibit elevated susceptibility to cybercrimes [9]. Various factors, including behavior, personality traits, mental state, online activity, response mechanisms, knowledge, and attitudes toward technology, contribute to individuals’ vulnerability to cybercrime [10, 11]. While the influence of internet usage on mental health has been extensively documented, a noticeable scarcity of information exists in the literature regarding internet-induced erotomania [12]. This gap underscores the need for further research and exploration into the potential connections between online activities and the emergence or exacerbation of specific psychiatric phenomena, such as erotomania.

Erotomania, also known as de Clérambault’s syndrome is a relatively rare, persistent delusional manifestation, characterized by an irrational belief that another person, usually of higher socioeconomic status, is romantically involved with the patient, despite minimal or no contact between them [13, 14]. This delusion typically involves morbid infatuation and the projection of the affected individual’s own attitudes onto the perceived “love object” [14]. It appeared officially as a diagnosis for the first time in DSM-III R. In both ICD-11 and DSM-5-TR. Erotomania is classified as a subtype of delusional disorder. In ICD-11, it is identified under the code MB26.04, with a definition emphasizing a persistent delusion involving the belief that a person of higher social or professional standing is romantically interested. In DSM-5-TR, Erotomania is similarly characterized by a persistent delusion, requiring a minimum duration of one month for diagnostic consideration [15, 16].

## Erotomania

### Historical perspective

Historical allusions to this phenomenon trace back to the writings of *Hippocrates*, *Erasistratus*, *Plutarch* and *Galen*, followed by descriptions and psychodynamic investigations undertaken by diverse scholars [17]. The Parisian physician *Bartholomy Pardoux* (1545–1611) studied the concepts of nymphomania and erotomania. In 1623, *Jacques Ferrand* referred to this syndrome as “*erotic paranoia*” in a treatise known as “*Maladie d’amour ou Mélancolie érotique*”. He also termed this condition “*erotic self-referent delusion*”. In 1848, *Sir Alexander Morrison* characterized it as a “*Delusion of Love*” highlighting its higher prevalence in women. *Gatian de Clérambault*’s seminal work in 1921 laid the groundwork for comprehending and acknowledging this distinct psychiatric phenomenon, terming it “*psychose passionelle*”. Subsequently, it has been referred to by various names such as “*erotic paranoia*”, “*erotic self-referent delusion*”, “*delusional loving*”, “*delusions of passion*”, “*phantom lover syndrome*”, “*psychotic erotic transference reaction and delusional loving*”, “*melancholie erotique*”, and “*amor insanus*” (“*insane love*”). Over the last few decades, the eponym “*de Clérambault’s Syndrome*” has garnered widespread acceptance within both the medical and psychiatric communities as a designation for this particular type of delusional disorder [18–20]. *Kraepelin*, *Bernard*, *Winokur*, *Kendler*, and *Munro* have added to the understanding of the disorder [17, 21–23]. *De Clérambault* identified two forms of erotomania: pure or primary, and secondary or recurrent [22, 24, 25]. The fundamental characteristic of the primary type of erotic delusion is the sole psychotic manifestation, unrelated to any other psychiatric or organic illness; hallucinations are absent; onset is abrupt; and the illness follows a well-defined, chronic course. The patient, typically a woman (subject), maintains the belief that she is the recipient of romantic interest from a man (object). Notably, in this scenario, the object must have initiated romantic feelings and made the first advances. Moreover, the object is perceived as being more deeply in love than the subject, and there may even be a lack of reciprocal feelings from the subject; the emphasis lies on the patient feeling loved rather than reciprocating the affection [26, 27]. *De Clérambault* additionally outlined other components of the syndrome, which he believed to be derived from the central belief [28]. The subject in de Clérambault’s syndrome typically has minimal prior contact with the object of love. An object is perceived as superior and unattainable due to various factors, such as high intelligence, exceptional beauty, fame, higher social status, or being married, among others [26, 27]. The subject, in the context of de Clérambault’s syndrome, is firmly convinced that the object cannot experience genuine happiness or completeness without her. In cases where the object is married, the subject may hold the belief that the marriage is not valid [26, 27]. *De Clérambault* outlined distinct stages in the evolution of erotomania: hope, resentment, and grudge, placing particular emphasis on the significance of the final phase [26, 27]. Initially, hopeful for a declaration of love, the subject’s persistent pursuit may lead to feelings of humiliation. Subsequently, the subject might develop resentment, potentially evolving into hatred and abusive behavior. The delusion of love may transform into persecutory delusion [26, 27].

Conversely, the secondary form manifests with a gradual onset, an ill-defined disease course, and the potential for substitution of the object of love. This form is associated with various mental disorders such as schizophrenia, bipolar disorder, major depressive disorder, and any subtype of dementia [23, 29–34], and may co-occur with Capgras syndrome, Fregoli syndrome, and Folie à Deux syndrome [35–38]. Notably, erotomania can also be secondary to physical or organic conditions. Reports highlighted cases associated with various factors, including meningioma, head trauma, convulsions, subarachnoid hemorrhage, pregnancy, HIV infection, Cushing’s disease, premenopause, alcohol or substance abuse (including cannabis), the use of oral contraceptives, amphetamines and antidepressants, mental retardation, and even orchiectomy [20, 23, 27, 34, 39–41]. Emotional abandonment and sexual inexperience may also have a significant effect on this delusion [42].

### Conceptual background

*Berrios and Kennedy*’s historical analysis provides valuable insights into the changing conceptual landscape of erotomania across different epochs [17]. *Kraepelin* characterized erotomania as “*compensation for life’s disappointments*” [43]. *Hart* (1921) referred to erotomania as “*Old Women Insanity*”, a condition in which delusion of persecution frequently manifests. In his posthumously published study in 1942 titled “*les psychoses passionelles*,” De Clérambault introduced the concept of “*sexual pride*” [28]. According to this concept, erotomania arises from a lack of affective and sexual input, becoming a means of satisfying an individual’s pride. *Segal* proposed that the erotomaniac delusion fulfills the patient’s need for love, representing the ultimate form of approval [44]. *Taylor* and others highlighted patients’ isolation, loneliness, and extreme dependence on others [45]. *Freud* viewed erotomania as a defense mechanism against unwanted homosexual urges, leading to feelings of paranoia, denial, displacement, and projection [46]. In 1967 *Trethowan* illustrated the social features of erotomania, he linked the patient’s past issues with relationships with parents to the current erotomania [47]. Various authors have delved into the psychodynamic etiology of this syndrome, suggesting that delusion serves as a gratification to individuals’ narcissistic needs [48]. The fundamental human desire for love is explored, particularly in the context of societal rejection, which can trigger the development of delusions. *Hollender and Callahan* present another compelling psychodynamic explanation, proposing that the development of an erotomanic delusion is rooted in an ego deficit [49]. According to their perspective, this deficit is shaped by an intrapsychic struggle wherein the individual grapples with a profound sense of feeling unattractive and unlovable, stemming from a narcissistic blow [48]. According to *Cameron*, erotomania is conceptualized as a manifestation of denied self-love that is projected onto another individual, specifically a man [50]. *Raskin and Sullivan* view erotomania as an adaptive function, that involves eliminating depression and loneliness following a loss [51]. The psychodynamic explanation provided by *Feder* adds a layer of complexity to the understanding of erotomania, suggesting that the delusional beliefs associated with romantic love may be intricately linked to early developmental experiences, and the psychological need for a sense of union and attachment, particularly with a maternal Fig. [52].

### Etiology

The true origins of this perplexing disorder remain largely elusive. Various explanations have been proposed for pure/primary erotomania, encompassing psychodynamic theories and neurophysiological factors such as visuospatial functioning deficits, limbic system lesions (especially in the temporal lobes), associative deficits, and impairments in cognitive flexibility and frontal-subcortical functioning. Although the etiology of primary erotomania has not yet been fully elucidated, neuroimaging, genetic studies, and insights from evolutionary psychopathology show potential for providing a more profound and comprehensive understanding of this complex condition. Previously there was a lack of evidence supporting the genetic role, but Gauld et al.‘s (2021) ground-breaking case report represents a pivotal study, the first one to propose a genetic molecular foundation for erotomania and to demonstrate familial aggregation. This study identified a family exhibiting a frameshift variant in the AUTS2 gene, shedding light on the potential genetic underpinnings of this complex psychiatric condition [53]. A neurochemical hypothesis suggests that the syndrome may result from a dopamine/serotonin imbalance, and it is posited that the interplay of environmental, psychological, pharmacological, and physiological factors may trigger erotomania in predisposed individuals [22, 26, 27]. Pure/primary erotomania is generally considered a chronic and refractory condition with a poor prognosis; however, there have been reports of cases showing a good prognosis [20, 26, 27]. Erotomania secondary to schizophrenia or schizoaffective disorder tends to have a worse prognosis, while erotomania secondary to affective disorders, such as bipolar disorder, appears to have a more benign course. In these cases, patients often exhibit a recurrent pattern of illness and maintain higher levels of functioning [20, 26, 27]. Some studies propose that delusions associated with this syndrome might develop as a coping mechanism in response to extreme stress or trauma [18, 23].

### Incidence and demography

Erotomania have been reported from various regions worldwide. The condition can manifest from adolescence to old age, and is not associated with any specific age group, race, culture, or socioeconomic status [18, 26, 27, 45]. Despite its prevalence, the exact incidence of erotomania remains unknown, but that of delusional disorder in general has been reported to be approximately 15 cases per 100 000 people per year, with a female/male ratio of 3/1. Historically, erotomania was believed to be more common in females, as it was once exclusively diagnosed in women. However, there are few case reports about male subjects, with males predominating in forensic samples. Some authors consider male erotomania to be more common than previously thought [45]. In general, the love object is of the opposite gender, but there have been reports of cases involving people of the same gender [23, 26, 27, 54]. Individuals with developmental disabilities may also be affected [23, 55]. It is possible that the incidence of erotomania is underestimated because erotomanic delusions may be considered as symptoms of other mental disorders.

Patients presenting with erotomania are typically described as socially isolated persons with little relational and sexual experience and persons inordinately preoccupied with their own needs and living a lonely life [23, 56]. Most authors describe unsatisfactory relationships between patients and their closest relatives, while some describe very close and dependent relationships with them, sometimes never separating from them, with the mother being the most important influence [45]. It is remarkable that erotomania can manifest discreetly over extended periods, often coming to light only when the individual engages in stalking or displays violent behavior towards the object of their affection. Risk factors for violence in erotomania include male sex, low socioeconomic status, multiple objects of love, and a history of antisocial behavior [23, 45, 54–58].

### Treatment

Erotomania requires active treatment and risk management as it can be associated with stalking and other offending behavior. In addressing erotomania, a multifaceted approach to treatment has been explored due to the intricate nature of the condition. Antipsychotic medication has proven effective in mitigating the intensity of delusions and controlling associated behaviors [26, 27]. For secondary erotomania, initial management focuses on addressing underlying organic or psychiatric illnesses. The comprehensive management of both primary and secondary erotomania entails a combination of pharmacological treatments, psychosocial interventions, and risk management strategies, especially considering the clinical experience, that monothematic delusions, such as erotomania tend to be quite resistant to different treatment modalities.

Historically, the antipsychotic medication pimozide has been widely used, particularly in certain countries such as the U.S. and Canada, despite limited systematic studies on its efficacy in treating this disorder [18]. Besides antipsychotic effect, potential adverse effects, such as hyperprolactinemia or decreased libido may also have clinical significance on patients with erotomania treated with antipsychotic medications, especially with the first-generation ones. Recent reports highlight positive therapeutic outcomes with second-generation antipsychotics (e.g. risperidone, clozapine), which offer improved tolerability compared to older agents such as pimozide, potentially enhancing patient acceptability and clinical outcomes [18]. However, there is a critical need for controlled clinical trials to explore therapeutic strategies for primary erotomania and related syndromes.

Presently, risperidone, especially at doses less than 6 mg/day, is the primary pharmacological intervention [18, 23]. Notably, patients with pure or primary erotomania exhibit a more favorable response to antipsychotic treatment than patients with other psychotic disorders [23, 26, 27]. Alongside pharmacotherapy, electroconvulsive therapy (ECT) has demonstrated efficacy, particularly when integrated with other treatment approaches [18, 23]. While individual psychotherapy has limited effectiveness, interventions involving family, social, and environmental factors may yield positive outcomes [20, 22]. In severe cases, temporary separation from the object of love may be necessary, potentially requiring hospitalization or legal measures. Intriguingly, evidence suggests that such separation could be the most efficacious treatment for erotomania [20, 26, 27].

## Aim

While the association between internet usage and the onset of erotomania is already documented [12], the concealed psychological hazards associated with online fraud, and its potential impact on the development of erotomania, constitute areas that have not yet received adequate research attention. In our clinical practice, we documented three patients who developed erotomanic delusional disorder as a result of online romance fraud. Because of delusion-induced family conflicts in two of these patients and the ensuing financial damage, one patient experienced a single suicide attempt, while the other had multiple suicide attempts. Among the three patients, only one gave consent for the disclosure of their case.

This case presentation illuminates a recently emerged phenomenon, induced erotomania due to online romance fraud, emphasizing the paramount importance of early recognition of this syndrome within the mental health patient population. The documented cases underscore the urgent need for increased awareness and intervention strategies to address the intersection of online romance fraud and the development of erotomanic delusional disorder.

## Case presentation

### Admission

A 70-year-old married Caucasian woman was admitted to our department following a suicide attempt by benzodiazepine ingestion, as a consequence of repeated conflicts with her husband. The patient revealed her persistent online communication and romantic involvement with a world-renowned musician in his thirties, a relationship spanning more than a year since her husband gifted her a smartphone.

### Present situation

The patient’s involvement began innocently, expressing admiration for the musician’s work through positive comments on various profiles featuring his art. Over time, the patient’s feelings intensified, perceiving the musician as talented, elegant, and sexually attractive. She persuaded her husband to attend the musician’s live concert, hoping for a personal encounter. Subsequently, she engaged in frequent, confidential chats on profiles using the musician’s image, receiving positive and encouraging messages. The conversations became increasingly personal, with the patient divulging significant amounts of information.

The patient received messages praising her extensively, creating a deep affection that she felt rejuvenated her life. This emotional involvement prompted her to enhance her appearance using cosmetics and undergo weight loss through dieting. To substantiate her claims, she asserted that the “musician’s secretary” once called her, arranging a telephone conversation during which he professed his love. The patient disclosed her feelings to her husband, who initially dismissed them.

As the online relationship deepened, the patient began receiving requests for significant sums of money, citing various reasons such as charitable causes or covering their phone bills. This led to escalating conflicts within the marriage, with the husband struggling to accept the contradictions of the perceived “love relationship” and expressing concerns about its dangers. The husband filed a police report without the patient’s knowledge, resulting in the cessation of fraudulent profiles. However, the fraudster resumed communication on a new profile, prompting the husband to transfer money in an attempt to stop the claims.

Despite these efforts, the fraudster held additional requests, intensifying her marital discord. The patient, fearing the loss of her “love,” became increasingly distressed. She accused her husband of obstructing her relationship, leading to threats of suicide. When her husband failed to respond, she ingested 30 tablets of 0.5 mg alprazolam.

### History

The patient, a retired cook, resides with her husband and reported feeling neglected by her family throughout her life. Her childhood and adolescence were uneventful, but she perceived familial neglect, exacerbated by her mother’s depression and dementia. Marrying a military officer at 17, her husband became her sole relationship. Over the past three decades, cumulative organ diseases have contributed to a depressive mood, decreased work capacity, and heightened isolation. Despite residing near her son and grandchildren, loneliness persisted due to infrequent visits, and her husband’s social engagement.

The medical history included bilateral deep vein thrombosis, diabetes mellitus with polyneuropathy, hypercholesterolemia, spondylosis, lumbar spondylarthrosis, osteoporosis, lumboischialgia, vertebrobasilar syndrome, and tinnitus. She took 50 mg of sertraline and 0.25 mg of alprazolam daily for dysthymia and anxiety, maintaining a compensated psychiatric status since the age of 55. No prior psychotic symptoms were noted.

### Diagnostics

Physical examination revealed moderate obesity, sensory disturbances associated with polyneuropathy in the lower limbs, mild cognitive impairment, poor thinking, depressed mood, emotional lability, slowed psychomotor activity, erotomanic delusions and suicidal thoughts. The patient reported loss of appetite and sleep disturbances.

Initial laboratory tests revealed elevated serum benzodiazepine levels and slightly increased hemoglobin A1C, cholesterol, and LDL-cholesterol values. Brain Magnetic Resonance Imaging (MRI) confirmed microvascular lesions (Fazekas I. stage).

After addressing benzodiazepine intoxication and improving affective symptoms, psychological tests (Minnesota Multiphasic Personality Inventory (MMPI), Structured Clinical Interview for DSM-5 - Personality Disorders (SCID-5-PD)) indicated a characteropathic mode of functioning with A- and C-cluster traits, psychotic and organic symptoms, limiting the perception of reality, insight, and judgment skills. Patient scored 27/30 points on the Mini Mental State Examination (MMSE) test and had 71/100 points on the Addenbrooke’s Cognitive Examination III (ACE-III) test.

Examinations revealed a diagnosis of mild cognitive impairment, delusional disorder of the erotomanic subtype, and organic affective disorder with a cerebral vascular background. We consider this case as an online romance fraud induced, secondary erotomania, on the basis of another psychiatric and organic disorder.

### Treatment

Treatment involved previous antidepressant medication (sertraline 50 mg/day) in combination with low-dose risperidone (2 mg/day), alongside supportive individual and group therapy. Despite the treating physician and psychologist presenting the patient with harsh facts, initially she remained ambivalent, and her erotomanic content persisted. After establishing an empathetic, accepting, and supportive relationship, we addressed emotional background, marital conflict, and previous negative events. Cognitive-behavioral therapy focused on processing the double-disappointment (deception and emotional loss) experience, while gradual feedback aimed to strengthen her insight into the unrealistic elements of the online “love relationship.” Couple-counseling resolved marital crises, leading to a more balanced relationship. Four weeks into treatment, dysthymia and anxiety resolved, psychotic symptoms improved, and the patient partially realized the fraud. Continuing psychopharmacological and supportive treatment on an outpatient basis was recommended.

## Discussion

In the early eighteenth century, erotomania was conceptualized as a general disease, the causal factor of which was thought to be unrequited love [17]. From the early nineteenth century to the beginning of the twentieth century, there was a conceptual transformation, with erotomania being recognized as a form of mental disorder primarily linked to unrequited love [17]. The evolution of this understanding continued into the early twentieth century and persists to the present day. In the contemporary context, erotomania is defined as a psychotic, delusional belief. Modern psychiatry postulates that the intricate interplay between stress, personality patterns; and environmental, genetic, psychological, pharmacological, and physiological factors may trigger erotomania in predisposed individuals.

The literature has recognized instances of erotomania induced by pre-Internet media (celebrity magazine, radio, TV, movie) referred to as “*Cinderella syndrome*” and more recently, social media networks have been implicated in psychotic disorders. With the widespread adoption of online communication and rapid technological advancements, it is predictable that romantic deceptions will become increasingly prevalent [59]. Faden and Prasad each separately emphasize the role of social media in individual case studies [12, 60]. The application of artificial intelligence (AI), such as Chat-GPT, breaks down linguistic barriers, making deceits more accessible, as romantic communication becomes possible even when the perpetrator is not fluent in the victim’s native language [61]. However, there is limited documentation on the impact of online romantic fraud on mentally impaired individuals [62].

In the present case, it becomes evident that the emergence of delusional symptoms was influenced by a combination of the patient’s personality traits, mild cognitive impairment with a vascular background, and ongoing life events (online romance fraud). Stressors associated with age-related changes, such as chronic physical illnesses, retirement, marital conflicts, familial estrangement, loneliness, and isolation, contributed to an identity crisis linked to this life-stage. The crisis was further intensified by the patient’s disharmonious personality traits and dysthymic mood disorders. Unfortunately, the patient lacked adequate coping and problem-solving mechanisms to navigate the crisis, and external assistance was not sought.

The intricate interplay of predisposing factors and emotional vulnerability may have laid the groundwork for the patient to become victim. Her low technical expertise and skills only heightened her vulnerability to cybercrime. Immature and ineffective defense mechanisms, such as projection, splitting, or denial, coupled with cognitive decline, led to a lack of insight into the unreality of the virtual relationship. Challenges in emotion regulation set the stage for a gradual progression toward erotomania, with delusions eventually dominating the individual’s thoughts and behaviors.

Repeated confrontations with the realities of her husband escalated their conflicts. These conflicts, combined with the fear of termination of the romantic relationship, may have contributed to the development of a regressive, narrowed mental state, ultimately culminating in a suicide attempt.

Given the complex interplay of individual and environmental factors underlying these processes, the treatment of emerging or worsening mental disorders necessitates the application of comprehensive bio-psycho-socio-spiritual perspective. In addition to psychopharmacotherapy tailored to psychological symptomatology (antidepressant, anxiolytic, or antipsychotic treatment), providing psychotherapeutic support is essential for processing the negative emotions (depression, grief) caused by deception.

Psychotherapeutic interventions should address two significant aspects. First, we should focus on the double trauma experienced by victims - financial loss and the dissolution of a perceived relationship. Second, acknowledging and addressing victims’ shame upon discovering fraud are vital. By integrating these considerations into the therapeutic approach, clinicians can enhance their effectiveness in helping individuals cope with the aftermath of online romance fraud and erotomanic delusions. The involvement of individuals in the patient’s environment (partner, children, other relatives, etc.) is crucial for treatment, aiming to address the patient’s relationship issues and alleviate social isolation. In addition, adequate psychoeducation is vital for drawing attention to the dangers of online communication, emphasizing the importance of not divulging personal information that could be exploited by online partners. Heightened caution is necessary when a “dear acquaintance” remains elusive and unwilling to engage in personal or video communication.

Recognizing that shame or distorted insight can be barriers to patient disclosure of victimization is vital. Establishing a safe, reliable, and supportive doctor-patient relationship is crucial for overcoming these barriers. Emphasizing the avoidance of victim-blaming is essential for a compassionate and effective approach. Exploring the patients’ personality traits and psychopathological symptoms is crucial, as they may increase the risk of becoming a victim. Factors such as dependent personality traits, content indicating unrealistic idealization, impaired situation awareness, problem-solving, and mentalization skills associated with cognitive decline, loneliness, isolation, and relationship or family issues can contribute to the risk of victimization. The presented case illustrates the role of these factors, as well as the stages of deception. Recognizing these factors is essential, as they increase the likelihood of preventing the worsening of unrealistic emotions and the development of severe psychopathological symptoms in the early stages of victimization.

## Conclusions

The presented case highlights the susceptibility of individuals with mental disorders to developing erotomanic delusions in the context of online romance fraud. It is crucial to monitor the online activity of such patients, particularly those with specific risk factors, as they are more likely to become victims. Identifying personality characteristics and psychopathological symptoms that elevate the risk of victimization is essential. These may include dependent personality traits, unrealistic idealization, cognitive function impairment affecting situational awareness, reduced problem-solving abilities, compromised mentalization skills, experiences of loneliness and isolation, and relationship or family problems.

In the course of treatment, avoiding victim blaming is paramount, and establishing a safe, trusting, and supportive therapeutic relationship is essential, which may also form a stable basis for adequate compliance with the treatment, including pharmacotherapy (antipsychotic medication). Psychoeducation should emphasize the risks associated with online personal data communication with strangers. Consultation with affected relatives is an integral component of psychotherapy.

These conclusions emphasize the importance of a comprehensive and multidimensional approach for both prevention and intervention strategies. By understanding the unique vulnerabilities of individuals with mental disorders, mental health professionals can contribute to reducing the incidence and impact of online romance fraud induced erotomania.

However, further comprehensive investigations into the concealed psychological risks associated with online fraud and its potential contribution to the development of erotomania are imperative. This call for in-depth research underscores the necessity of understanding the intricate relationship between online fraud and the emergence of erotomania, with the aim of informing preventive measures and tailored interventions within the realm of mental health.

## Data Availability

No datasets were generated or analysed during the current study.

## Abbreviations

ICD-11: International Classification of Diseases and Related Health Problems 11th revision
DSM-5: Diagnostic and Statistical Manual of Mental Disorders, 5th edition
